# "The Hospital" Medical Book Supplement.— No. IX

**Published:** 1908-07-18

**Authors:** 


### "THE HOSPITAL" MEDICAL BOOK SUPPLEMENT.—No. IX.

CONTENTS.

**Surgery—**
Grandular Enlargements and Other Diseases of the Lymphatic System ... 423
Health at its Best v. Cancer ... 423
The Operations of General Practice ... 423

**Medicine—**
A Manual of the Practice of Medicine ... 424
A Manual of the Practice and Theory of Medicine ... 424
International Clinics ... 425

**Clinical Pathology—**
Clinical Methods: A Guide to the Practical Study of Medicine... 425
"The Heart and Sudden Death" ... 425

**Miscellaneous Items—**
The Experimental Prophylaxis of Syphilis ... 426
The Hair and Its Diseases, including Ringworm, Greyness, and Baldness ... 426
How to Nurse the Patient ... 426
Hay-Fever, Hay-Asthma: Its Causes, Diagnosis, and Treatment 426
Guide-books for the Holidays ... 426

### SURGERY.

Glandular Enlargements and Other Diseases of the Lymphatic System. By Arthur Edmunds, F.R.C.S. Pp. viii.+230. 19 figs. (Henry Frowde, Hodder and Stoughton, Oxford and London. 1908. Price 7s. 6d. net.)

This handy volume is one of the series of Oxford Medical Publications—a fact which should vouch for its general standard. As the author states, an exhaustive treatise upon the lymphatic system would entail a review of almost the whole of medicine and surgery, for there are few diseases in which the lymphatic system does not play some part. He confines himself to those affections in which trouble in the lymphatic vessels or glands constitutes the essential feature of the disease. We may say that he does not reach the confines even of these.

The printing is good. The illustrations have been carefully prepared, but we are disappointed with them, for they must have added much to the cost of the book without conveying a great deal of information to the reader who does not already understand the conditions which they illustrate. To those who know, the figures are unnecessary; to those who do not know, many of them must seem obscure.

We are bound to admit that the book disappoints us in many ways. If its title had been "Glandular Enlargements from a *Surgical* Point of View" it would have more clearly expressed the character of the volume. The reader will find little or nothing about lymphatic glandular enlargements in German measles for example; lymphatic leukæmia is barely mentioned, and there is little or nothing about the assistance of blood-examination in diagnosis. Here and there it may be stated that "examination of the blood is extremely important," but practically no details as to what the blood-counts show in different conditions are given. Diseases of everyday importance, such as enlargement of cervical glands due to pediculosis capitis or to scarlet fever, are not mentioned in the index. We have not found any account of the diagnostic value of enlarged left supra-clavicular glands in cases of abdominal malignant disease; Handley's operation for the relief of lymphatic obstruction by continuous strands of silk does not seem to be mentioned either. In short, the book is written mainly from the surgical point of view, and even from that it leaves a great deal unsaid.

Health at its Best *v.* Cancer. By Robert Bell, M.D., F.F.P.S., formerly Senior Physician to the Glasgow Hospital for Women. Pp. 320. (T. Fisher Unwin, London and Leipsic. 1908. Price 5s. net.)

This book is apparently written for the general public rather than for the medical profession. It is one of many that have been published upon the text "Errors of diet and of the general way of living are the cause of cancer." We have always allowed that there may be something in this supposition, and we should be the last to pooh-pooh the notion forthwith; it is a matter which needs the most careful investigation. Nevertheless, we are sincerely sorry to see works published upon the subject imbued from start to finish with the one idea that dietetic and allied errors are a prime cause of cancer. They may be; but we do not know that they are. It is misleading to the public to say that "cancer is Nature's protest against over-indulgence of the appetite and the persistent neglect of, or disobedience to, those hygienic laws which she has enacted." Dr. Bell seems to be so certain in his own mind that cancer is not a local disease, but a local manifestation of a generally diseased state of the blood, that he strongly deprecates any operative measures for its cure. "Why, the very nature of the disease contra-indicates operation. . . . Were it a local disease and nothing more, were there no blood contamination lurking behind, then surgical interference would be warranted—nay, more, it would be wrong not to resort to it. But when we know for a fact that primarily this fluid is the nidus of the disease . . . it does not require a high intelligence to conclude that temporary, and often very transitory, relief to a symptom can in no instance succeed in removing the disease, of which the local manifestation is only an outcrop." "The disease in a very short space of time reappears with renewed virulence in the mutilated part, and speedily rages with increased potency, the progress of the local mischief being much more rapid and painful than if the parts had been left alone."

Our readers will see from the above the kind of views that are to be met with in the book. The work may frighten some members of society into living healthier lives, but we do not see what other good result can come of its publication.

The Operations of General Practice. By E. M. Corner, M.C., F.R.C.S., and H. I. Pinches, M.B. (London: Henry Frowde and Hodder and Stoughton.) 2nd edition. Revised and enlarged 1908. Pp. 338. Price 15s. net.

The very favourable impressions which we formed of this work less than a year ago have been quickly confirmed and verified by the exhaustion of the first edition. In the present stage of the evolution of surgery it is not likely that the operations of general practice will ever remain long unamplified, and it is not surprising to find that the additional chapter now added includes twenty proceedings not dealt with in the first edition. Of these that describing the radical treatment of hydrocele by eversion of the tunica vaginalis is the most likely to be of value and interest to hospital officers and practitioners, though many of the remainder are distinctly welcome. The description of a new method for dealing with hæmorrhoids is a trifle vague and difficult to understand, but otherwise the same clear and explicit style is observed which has no doubt contributed largely to the popularity already attained by the volume.

### MEDICINE.

A Manual of the Practice of Medicine. By Frederick Taylor, M.D., F.R.C.P. Eighth Edition. (London: J. and A. Churchill. 1908. Pp. 1,111. Price 16s. net.)

When a text-book on a subject as large as that of medicine reaches an eighth edition within eighteen years of its original publication, it is worth while to cast about for the secret of its success and popularity. The quality above all others which is pre-eminent in "Taylor" is perhaps best expressed by the adjective businesslike. The author has no fads to air, no superfluity of rhetoric to show off, but gets to the root of matters in as clear and simple phraseology as possible. It is this which especially recommends him to the fifth-year student, who feels he cannot afford to "skip" and is fairly confident that a general acquaintance with the contents of this text-book will carry him safely through any of the qualifying examinations in medicine. It is claimed in the preface that the book is intended both for the student and the practitioner; but though it is the most useful text-book one could put into the hands of the former, it is perhaps for that very reason of somewhat less value to the latter. A more practical note is struck throughout than in many works on medicine designed for students, yet the general impression on re-reading the old favourite after an interval of some years is that it is not quite full enough for an intelligent practitioner in the matters of diagnosis and treatment. This defect is probably inseparable from the compression of so enormous a subject into so compact a form, and encyclopædic works of reference or a multiplicity of special monographs, which seem to be the alternatives, are admittedly costly luxuries.

The sections to which a critical eye is first directed in reviewing a text-book of medicine of long-established fame are those which deal with the most recent advances: in which one may note whether the book is adequately kept up-to-date, in fact. There is little to complain of in this respect; the new opsonic and other bacteriological methods of investigating disease are described briefly, and the claims made on their behalf are indicated without endorsement, an attitude which is both fair and prudent. In those diseases in the treatment of which surgery has come to the front Dr. Taylor is perhaps a little less abreast of the times: no one looks for details of surgical operations in a work on medicine, but the sections on intussusception and appendicitis, for example, show a conservative tendency which one would gladly see eliminated. There are occasional slips upon which it would be almost ungracious to dwell in dealing with a work of less authority, but cannot be passed over altogether in this case. Thus we find acute rheumatism classed in a list of disorders whose bacteriology is certain: whereas a few lines further on rheumatic fever is put down among those whose bacteriology is uncertain. In the section on malaria it is stated that negroes are less insusceptible to the disease than white men, which is possibly not what the author means: in this account (and indeed in dealing with other tropical diseases), modern research is fully recognised, but in the matter of ætiology the appearance is given of the present knowledge having been dovetailed into paragraphs on the older aerial views. In the prophylaxis of malaria it is recommended that two to five grains of quinine should be given three times a day, which is certainly too much: on the next page one finds five grains a day advised, with attention to mosquito curtains and the like, which is a much better scheme of prevention. The pages on intestinal worms are perhaps less good than most of the others, and of some of these parasites which, though uncommon in England, are not unknown, there is no mention at all.

The eight skiagrams of pulmonary and cardiac conditions which are now introduced have been well chosen, and increase the utility of the book. Indeed, the whole subject of intrathoracic disease is handled about as well as it is possible to do in the allotted space: there is scarcely a wasted word, and everything that a student can possibly be expected to know is fully dealt with. Heart block, for instance, is described, and the diagnosis and treatment of phthisis has been most thoroughly revised and added to. It has always been a strong point in Dr. Taylor's manual that skin diseases are briefly described, and this part of the work now runs to seventy-five pages. Like the other sections, it has been well edited, and one finds, for example, a satisfactory account of the *x*-ray treatment of ringworm. The advantage of including these diseases has always seemed to us that most students neglect the subject both theoretically and practically: they will not trouble to read a separate monograph on dermatology and so they lose interest in any skin cases that they may see; by describing these in his text-book Dr. Taylor not only enhances its usefulness, but also renders real service to medicine and medical students. We do not think we can sum up better than by expressing the wish that as good a text-book of similar size on surgery were in existence.

---

A Manual of the Practice and Theory of Medicine. By Sir William Whitla, M.A., M.D., LL.D., Senior Physician to, and Lecturer on Clinical Medicine at the Royal Victoria Hospital; Professor of Materia Medica and Therapeutics in Queen's College, Belfast. In two volumes. (London: Henry Renshaw. Pp. 932 × 968. Crown 8vo. Price £1 12s.)

This work, which is really a Dictionary of Medicine rather than a Manual of Medical Theory and Practice, will be found a useful addition to the practitioner's library. The title, as the author explains, has been determined by a desire to avoid confusion with his well-known "Dictionary of Treatment," to which it forms an excellent introduction and companion. The work has been divided into two octavo volumes, each with a complete index to both; and within this compass Sir William Whitla includes a vast quantity of information, arranged and written with much literary skill. The aim of the work is to afford to the busy practitioner, in the most convenient and condensed form, a series of independent articles upon the different diseases (including those of the skin) which fall under the province of the physician; and the author has succeeded very well in accomplishing his object. The dictionary form of arrangement, while it has many advantages from the point of view of him who wishes to read up a particular subject in the shortest space of time, has also certain disadvantages, notably that of inevitable repetition. The general standard of the many articles which are herein contained is remarkably high, and the short résumés of the ætiology and pathology of the various diseases are clear and up-to-date. The subject of treatment, although ably summarised—as is to be expected from Sir William Whitla—is reduced to the barest minimum, and, to use the book to the best advantage, it should be read in conjunction with the author's "Dictionary of Treatment." We have consulted the manual on a variety of topics, and have been struck by the lucidity and completeness of the descriptions. The author's views do not in every instance coincide with those which we accept, but this would be almost impossible, even if it were altogether desirable, in a work which sets out to include, in small compass, the whole vast field of medical practice. The prominence of the author's individual point of view is, indeed, one of the most attractive features of the manual, and adds greatly to the interest of many of the articles. The repetition of the complete index at the close of each volume renders reference easy, and there is a very complete arrangement of cross-references in the index to facilitate inquiries into the subject of differential diagnosis. We think that in a work of this scope and size the author has done wisely to exclude statistics, and confine himself to the broad principles which may be deduced from them. There is much clinical wisdom and sound practical advice scattered throughout these two volumes, and set forth with point and brevity. The article on Phthisis is a good instance of the author's method and style, and will be found very helpful. As an example of condensation without obscurity or inelegance, we might point to the short article on Dyspnœa, which the candidate for examination would do well to study. Broncho-pneumonia, to take a subject haphazard, is dealt with in six pages, and yet the clinical and pathological descriptions are very fairly complete, and the directions for treatment, if brief, are at least practical and suggestive. The sections dealing with the varied aspects and manifestations of heart disease are skilfully arranged and of uniform literary excellence. We have said enough to indicate that these two handy volumes by Sir William Whitla are likely to attain considerable popularity among practitioners of medicine. It is only necessary to add that the printing and paper are good, and the arrangement of type clear and convenient for reference. There are no illustrations or diagrams.

International Clinics. Vol. I. Eighteenth Series. (Lippincott, Philadelphia and London. 1908.)

This volume contains articles touching upon many branches of medicine. They are for the most part unsatisfying, and not seldom so superficial and sketchy that one is amazed that publishers should have given them the dignity of a binding, when they would have been more suitably accommodated between the paper covers of a weekly journal. The pretentious titles of some of the contributions are so ludicrously disproportionate to the meat in them that the natural indignation which the reader at first experiences yields presently to a sense of the ridiculous. For example, under the heading "Medicine" there appears a paper entitled "The Para-typhoid Fevers." It consists of four pages and a chart, and deals in the main with the record of a single case. Dr. Fordyce's article upon the value of the opsonic test and upon that of vaccine treatment in some infective conditions is the result, no doubt, of a deal of work. But its construction is so hopelessy confused as to leave obscured any new thing which there may be in it. We may admit that he knows what he is talking about, but we dare swear his readers will not be in the like fortunate position. Dr. John B. Deever dilates upon the diseases of the gall-bladder after the fashion of an elementary text-book, and a bad one at that. But there are some good things. For example, Dr. Lawrason Brown has written a really valuable and practical essay upon the sanatorium as a means for the treatment of pulmonary tuberculosis. The editors have done well to open the ball with Dr. Brown's article. Dr. Sommerville, dealing with mucous colitis in a short paper, accepts the view that the disease depends upon a deficiency of an anti-coagulant of mucus—and, incidentally of blood and milk—which is a normal ingredient of healthy bile. This hypothesis, or our dull conception of it, leaves us wondering how it is that the patient's mucus clots upon the walls of his large intestine while his small intestine remains unscathed. But at least Dr. Sommerville is readable, if not very illuminating. Dr. Rudolf contributes an interesting study of the normal temperature of the body, and Sir Dyce Duckworth one upon textural proclivities and the personal factor in disease. We wish more power to Sir Dyce's elbow, for his cause needs champions badly. Dr. Frank is bold enough to say he does not believe in purely functional disorders of the stomach; which means, we suppose, more gastro-enterostomies for neurasthenic dyspeptics—poor creatures. We are not qualified to appraise Dr. Deaderick's paper on the ætiology of hæmoglobinuric fever, but it appears to be a laborious and thoughtful piece of work. The last hundred pages of the book are given up to an indexed synopsis of the literature of medicine and surgery for the past year. It supplies a much-needed, though but partial, justification for the appearance of the volume.

### CLINICAL PATHOLOGY.

Clinical Methods: A Guide to the Practical Study of Medicine. By Robert Hutchison, M.D., F.R.C.P.; and Harry Rainy, M.D., F.R.C.P. (Ed.), F.R.S.E. Cassell and Co., Ltd., London. Fourth Edition. 1908. Pp. xv. + 632, with 11 coloured plates and 148 figures in the text. Price 10s. 6d.

This work is so familiar and so excellent that little need be said about it here beyond calling attention to the fact that a new edition has appeared, with several additions and alterations, especially in the chapters on the alimentary system, the blood, the urine, the nervous system, and clinical bacteriology. The original scheme of the work has been adhered to. To those who do not already know it, one may say that the book deals with almost all the clinical methods of examining both patients themselves and their secretions, excretions, and so forth. From the beginning at "case taking" the reader is conducted through every stage of physical examination, from inspection, palpation, percussion, and auscultation to testing the electrical reactions of the muscles; pulse tracings receive a section to themselves; the methods of testing urines, examining blood, sputum, pus, and so forth are fully dealt with; there is a special chapter upon clinical bacteriology; and a useful appendix upon weights and measures and the formulæ for solutions. The book contains an immense amount of the information required by medical students; the only question is whether this information is not too much for a single volume.

"The Heart and Sudden Death." By Theodore Fisher, M.D., F.R.C.P. London: The Scientific Press, Ltd. Pp. 53. Figs. 14. 1908. Price 2s. net.

Disease of the heart discovered during life comparatively rarely ends in sudden death, with the exception of aortic incompetence. Sudden death, on the other hand, in those who have hitherto been regarded as healthy, and in whom poisoning or violence can be excluded, is due to some heart affection in a great many cases. The little book before us gives an admirable description of the various cardiac lesions that may be found. The information is based upon the results of 2,500 autopsies made by the author, and every practitioner will find it useful in connection with the Coroner's court. We hope that Dr. Theodore Fisher will some day extend his work so that it may be entitled "Sudden Death from Natural Causes," including not only cardiac lesions, but also all the various other affections of the viscera that may lead to the same ending.

### MISCELLANEOUS ITEMS.

The Experimental Prophylaxis of Syphilis. By Dr. Paul Maisonneuve. Translated by F. L. de Verteuil, M.B., R.N. (Bristol: J. Wright and Company. 1908. Pp. 102. Price 4s. net.)

Two years ago the author of this monograph, then a medical student, submitted himself to inoculation at the hands of MM. Roux and Metchnikoff with active virus from two patients suffering from local and constitutional syphilis. An hour after the inoculation the lesions were treated with a strong calomel ointment. He escaped infection, and now publishes in a thesis the exact account of the experiment and of the careful way in which it was controlled, together with a history of all previous work in this direction and a *résumé* of the latest investigations. He takes particular care to formulate his deductions clearly, and has been well served in this respect by his translator. It goes without saying that no syphilologist can afford to overlook this work; but the general practitioner also is so much concerned with the question that neither should he neglect to be familiar with it. On that account it is perhaps to be regretted that a somewhat lower price has not been put upon this publication.

The Hair and Its Diseases, including Ringworm, Greyness, and Baldness. By David Walsh, M.D.Edin. Second Edition. (London: Baillière, Tindall, and Cox. Pp. viii+94. Illustrations, 5. 1908. Price, 2s. 6d. net.)

This booklet is not a monograph upon the hair, but an elementary handbook dealing with its physiology and commoner diseases in simple language. The first edition has been out of print for some years; the second has been re-written and a chapter upon affections of the nails and their treatment has been added. We cannot help thinking that the handbook would have been much more useful to practitioners if it had dealt thoroughly with the question of *x*-rays in depilation and in the treatment of sycosis and ringworm. There is no doubt whatever that the *x*-ray treatment of ringworm is rapidly superseding all other methods of curing the disease; it is disappointing to be merely told so without being given details as to the method of procedure, the necessary precautions, the causes of failure in some cases, and so on.

How to Nurse the Patient.—By many authors; edited by J. C. Wilson, M.B. (Glasgow and Edinburgh: Wm. Hodge and Co. Pp. 324. Illustrated.)

This book on nursing contains the work of seven ladies and two gentlemen, but the task of arrangement and supervision has been so conscientiously performed that the homogeneity of the result is in no way impaired. It is published for the benefit of the building fund of the Blantyre Cottage Hospital. In the preface it is claimed that methods of nursing are alone dealt with, and that treatment is to be left to the medical man. While recognising the practical character of the teaching as to nursing, it must be noted that the claim referred to is not one which we can admit. Thus to find cocaine and opium set down, and in liberal dosage, as drugs which should rarely (!) be resorted to except by direction of a medical man, is somewhat disconcerting; and, on reading a little further, to discover that a 2-per cent. solution of the former drug "will relieve pain and discomfort in the eye," without any indication as to the danger of a nurse or any other non-medical person thus meddling in eye-diseases, deprives us of much of the energy with which we might otherwise recommend the book. In fact, the medical excursions are unsatisfactory in other ways: it is always difficult to see what value snippets of incomprehensible medical and surgical aphorisms can have for a nurse, but when we read that a sharp pain below the knee is often premonitory of cerebral hæmorrhage, and that pain in the region of one kidney may be due to the presence of a stone in the other, we really wonder what object the statements are intended to serve by those who pen them. The remarks on headache are equally unsatisfactory, for the only form of this complaint in which the assistance of a practitioner is advised is that alleged to arise from the presence of intestinal worms.

Hay-Fever, Hay-Asthma: Its Causes, Diagnosis, and Treatment. By William Lloyd, F.R.C.S. Pp. 101. (Henry J. Glaisher, London. Second edition. 1908. Price 4s. 6d. net.)

The subject-matter of this work is exactly summarised by its title, and the book can be thoroughly recommended. There is a good epitome of the opinions held on the exciting causes of hay-fever, with illustrations of the various grasses whose pollen is most productive of the complaint in this country. Predisposing causes are discussed, and then follow chapters upon the symptoms and treatment. Many useful and practical suggestions are given under the latter heading. The book finishes with a clinical and therapeutical account of twenty-six illustrative cases. The type is large and clear, the style is clear and concise, and there is a very fair index.

**Guide-books for the Holidays.**

Nelson's sixpenny guides are a new departure in cheap guide-books. Each volume contains a number of beautiful plates, a large map of the district in three colours, and several smaller maps and plans. The books are handy for the pocket, and are neatly and tastefully bound. Brighton, Scarborough, Aberystwith, Torquay and Exeter, York, Falmouth, Ilfracombe, and Hastings are each dealt with in a single sixpenny volume. Nelson's "Guide to Paris," uniform with the others, is 9d., or 1 franc.

**Lewis's Library Catalogue.**

We have received from Mr. H. K. Lewis, 136 Gower Street, and 24 Gower Place, London, W.C., a copy of the "Catalogue of Lewis's Medical and Scientific Circulating Library," revised to the end of 1907. This will be found of considerable service to medical men and students who wish to have a handy bibliography of current medical and scientific literature; while to the subscriber to Lewis's Library it will prove invaluable. The catalogue is well printed and strongly bound, and consists of 500 pages, the last 190 of which are devoted to a classified index of subjects, with the names of those authors who have treated upon them. The price of this useful work of reference is 5s. net to non-subscribers, and 2s. net to subscribers to the Library.

**A Handbook of London Medical Schools.**

Under the direction of the Conference of Deans of the Metropolitan Schools of Medicine, an illustrated handbook, entitled "Medical Education in London," has recently been issued. The Conference of Deans has had especially in view the needs of visitors from abroad to the Franco-British Exhibition. Not only are the principal medical schools dealt with, but reliable information is provided concerning all the special hospitals, asylums, laboratories, and research institutions. The volume will be found most interesting and instructive. It is published by Ash and Co., Ltd., Henry Street, Southwark, S.E.

